# Bortezomib

**DOI:** 10.4103/0971-5851.76199

**Published:** 2010

**Authors:** Senthil Rajappa

**Affiliations:** *Department of Medical Oncology, Indo American Cancer Hospital and Research Institute, Banjara Hills, Hyderabad, India*

## INTRODUCTION

Bortezomib is a reversible inhibitor of the 26S proteasome, a protein complex that degrades ubiquitinated proteins. Proteins entering the proteasome are stripped of their ubiquitin, and subsequently degraded through catalytic activities within the core of the proteasome. The compound bortezomib (PS-341) contains a boronate moiety linked to a dipeptide and has exceedingly high affinity, specificity, and selectivity for catalytic activity of the proteasome.

Nuclear factor-κB (NF-κB) is a transcription factor that is retained in the cytoplasm when it is bound to an inhibitory partner protein, IκB. When IκB undergoes regulated serine phosphorylation, it is ubiquitinated and degraded in the proteasome. The released NF-κB moves to the nucleus, where it induces the transcription of genes whose protein products block cell-death pathways, promote cell proliferation, and regulate the expression of adhesion molecules. Inhibition of IκB degradation by proteasome inhibitors keeps NF-κB in the cytoplasm, thereby preventing it from acting on nuclear DNA.

**Figure 1:**
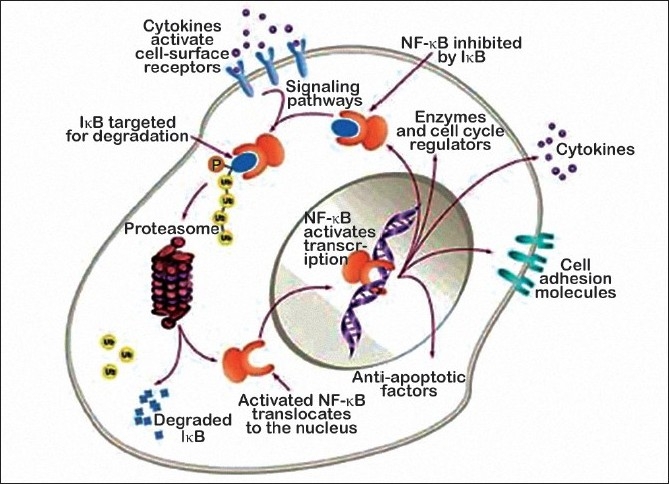
Mechanism of action of bortezomib

Modification of IκB also results in the sensitization of tumor cells to chemotherapeutic agents and radiation. However, the inhibition of the proteasome has other consequences, including decreased activation of the mitogen-activated protein kinase pathway and upregulation of p53 and the cell cycle inhibitor p27 [Figure 1]

Bortezomib has a distribution half-life of 10 minutes with wide interpatient variability in concentration. It is metabolized by oxidative deboronation via CYP 3A4 and 2C19. It has a terminal half-life of 9–15 hours.

## INDICATIONS

Multiple myeloma and relapsed mantle cell lymphoma.

## ADVERSE EVENTS

Peripheral neuropathy (36–37%, severe 8–14%): This is a common and often dose-limiting side effect. It is predominantly sensory, although mixed sensory–motor and autonomic neuropathy are also known to occur. Feet are affected more often than hands. The mechanism underlying bortezomib-induced peripheral neuropathy is not known. Patients with baseline symptoms are at a greater risk of developing severe neuropathy. Early detection and appropriate dosage adjustments may prevent development of severe neuropathies.

Thrombocytopenia (35–43%): Patients receiving bortezomib experience a median 60% decrease in platelet count regardless of initial baseline platelet count. The onset of thrombocytopenia most commonly occurs after cycle 1 or 2 and continues throughout therapy, with no evidence of cumulative thrombocytopenia. Platelet counts typically reach a nadir on day 11 and rise to a normal count by day. The mechanism underlying bortezomib-induced thrombocytopenia is unclear and is not secondary to bone marrow injury or thrombopoietin inhibition. Hence, supportive care rather than discontinuation of bortezomib therapy may be appropriate. There have been reports of GI and intracerebral hemorrhage in association with bortezomib-induced thrombocytopenia.

Diarrhea (55–58%): Diarrhea is a common side effect and can be severe in 7–8% of patients.

Hypotension: Hypotension occurs in up to 12% of patients receiving bortezomib. Risk factors include history of syncope, concomitant use of medications known to lower blood pressure, and dehydration.

Liver failure: Rare cases of acute liver failure have been reported in bortezomib-treated patients on multiple concomitant medications and with serious underlying medical conditions. Other reported hepatic events include asymptomatic increases in liver enzymes, hyperbilirubinemia, and hepatitis. These changes may be reversible upon discontinuation of bortezomib.

Hyperuricemia may result from cell lysis by cytotoxic chemotherapy and may lead to electrolyte disturbances or acute renal failure. It results most likely with highly proliferative tumors of massive burden, such as leukemias, high-grade lymphomas and myeloproliferative diseases. The risk may be increased in patients with pre-existing renal dysfunction, especially ureteral obstruction.

Pulmonary: Cough (17–21%), dyspnea (21–25%)

Pain: Limb pain (15%), musculoskeletal pain (10%)

Musculoskeletal: Arthralgia (15–28%), muscle cramps (24%)

Neurological: Dizziness (14–21%), headache (28%), insomnia (18–27%), anxiety (14%)

Dermatology: Rash urticaria (24–28%)

Hematology: Anemia (26–32%, severe 9–10%), neutropenia (19–24%, severe 14–16%)

Constitutional: Fatigue (61–65%), fever (31–36%), insomnia (18%), rigors (11–12%)

## SPECIAL PRECAUTIONS

The drug is contraindicated in patients who have hypersensitivity to bortezomib or boron or any of the constituents. All patients will have to be tested for HbS Ag and HbC Ab before starting the drug. Those who are positive should be on lamivudine with frequent liver function monitoring during the entire course of bortezomib administration. Patients also need to be on prophylaxis for varicella zoster reactivation.

At the onset of any grade 4 myelosuppression, the drug is immediately discontinued and the dose should be decreased by 25% for subsequent cycles. It is to be used with caution in renal impairment. In patients with liver dysfunction, the dose is 0.7 mg/m^2^ for those with serum bilirubin >1.5 × upper limit of normal (ULN) and any elevation of AST. For subsequent cycles, the dose can be escalated to 1 mg/m^2^ depending upon the tolerance.

For grade 1 neuropathy with pain and grade 2 neuropathy, the dose is decreased by 25%. The drug is stopped in those with moderate (grade 2 painful and grade 3) neuropathy and resumed at 0.7 mg/m^2^. For those with grade 4 symptoms, the drug is permanently discontinued. Similarly, appropriate dose adjustments have to be made for patients developing diarrhea.

The drug is contraindicated in pregnancy and lactation.

## DRUG INTERACTIONS

Bortezomib may inhibit CYP450 isoenzyme 2C19 and increase the levels/effects of CYP2C19 substrates. Bortezomib is a substrate mainly for CYP3A4 and CYP2C19. Patients receiving bortezomib with inhibitors or inducers of CYP3A4 and 2C19 should be monitored for potential toxicities or reduced efficacy associated with concomitant use. Bortezomib efficacy severely decreased, when concomitantly used with green tea or preparations made from green tea.

Bortezomib is supplied as 1, 2 and 3.5 mg sterile preservative free lyophilized powder for injection. It is administered as a slow IV push over 3–5 seconds. It is administered at a dose of 1.3 mg/m^2^ on days 1, 4, 8, 11 every 3 weeks.

## OVERDOSAGE

Overdosage with as little as twice the recommended dose has been associated with the acute onset of symptomatic hypotension and thrombocytopenia with fatal outcomes. In the event of an overdosage, monitor vital signs and provide supportive care to maintain blood pressure and body temperature.

